# The Association of FIT-Based Colorectal Cancer Screening with Earlier Disease Detection: The Romanian Experience

**DOI:** 10.3390/cancers18172815

**Published:** 2026-08-31

**Authors:** Catalin-Andrei Dutei, Gabriel Richiteanu, Florin Andrei Grama, Gabriel Becheanu, Bogdan Cotruta, Teodora-Ecaterina Manuc, Andreea-Elena Chifulescu, Mircea Manuc

**Affiliations:** 1 Department of Gastroenterology, “Carol Davila” University of Medicine and Pharmacy, 050474 Bucharest, Romania; catalin-andrei.dutei@drd.umfcd.ro (C.-A.D.); teodora.manuc@umfcd.ro (T.-E.M.); andreea-elena.grigore@drd.umfcd.ro (A.-E.C.); m_manuc@yahoo.com (M.M.); 2 Department of Surgery, “Carol Davila” University of Medicine and Pharmacy, 050474 Bucharest, Romania; 3 Department of Surgery, Coltea Clinical Hospital, 030171 Bucharest, Romania; 4 Department of Gastroenterology, Fundeni Clinical Institute, 022328 Bucharest, Romania; gbecheanu@yahoo.com (G.B.); bogdancotruta@gmail.com (B.C.); 5 Department of Histopathology, “Carol Davila” University of Medicine and Pharmacy, 050474 Bucharest, Romania

**Keywords:** colorectal cancer screening, colorectal cancer, adenoma detection rate, opportunistic colonoscopy, early detection rate, national screening programs

## Abstract

**Background/Objectives:** Given the increasing burden of colorectal cancer (CRC), effective screening strategies are essential for early disease detection. As a primary endpoint, this study aimed for the assessment and comparison of two different diagnostic pathways in the Romanian population: population-based screening using the fecal immunochemical test (FIT) and opportunistic, symptom-driven, colonoscopy. **Methods:** A retrospective observational study was conducted in two groups comprising a total of 236 patients from an average-risk population across Romania. Group A consisted of patients diagnosed through a CRC screening pathway based on FIT, whereas group B included patients diagnosed following opportunistic colonoscopy performed in routine clinical practice. Patients were retrospectively analyzed according to the tumor–node–metastasis (TNM) classification, with early- versus late-stage disease defined on the basis of overall TNM stage. Overall stage, tumor (T) stage and lymph node (N) stage were additionally analyzed as ordinal outcomes. Binary and ordinal logistic regression models were adjusted for tumor location. **Results:** The odds of detecting CRC in stage I were 2.76-fold higher in the screening group than in the opportunistic colonoscopy group. For advanced stage disease, the difference did not show statistical significance. Analyses, stratified by tumor location, identified a statistically significant association for left-sided colon and rectal tumors. The odds of detecting a T1 tumor were 6.34-fold higher in the screening group than in the opportunistic colonoscopy group. Regarding lymph node involvement, the odds of detecting N0 disease were 2.03-fold higher in Group A than in Group B. **Conclusions:** Compared with opportunistic diagnosis, the screening pathway was associated with a higher likelihood of detecting CRC at an earlier stage, including tumors with limited local invasion and no lymph node involvement. These findings support the potential benefit of population-based CRC screening for earlier disease detection, although they should be interpreted in the context of the observational study design.

## Simple Summary

Given the increasing burden of colorectal cancer in the general population, effective screening programs are essential for the detection of early-stage disease and may provide substantial benefits, compared with opportunistic diagnosis, based on symptom-driven medical consultations and diagnostic procedures, including colonoscopy. In this observational study conducted in Romania, the distribution of tumor stage, local invasion and lymph node involvement was assessed in two distinct groups comprising a total of 236 patients. Comparative analysis revealed a statistically significant endpoint: there is a higher probability of detecting colorectal cancer at an early stage and without lymph node involvement through a screening pathway in the general population, rather than by opportunistic diagnosis. These findings should, however, be interpreted in the context of the limitations inherent to the non-prospective, non-randomized study design.

## 1. Introduction

Colorectal cancer (CRC) represents a major global health burden, ranking third in incidence and second in cancer-related mortality worldwide, with an estimated 1,926,425 new cases (9.6% of all cancer cases) in both sexes combined, according to the most recent Global Cancer Statistics report (2022) and accounting for approximately 904,019 deaths (9.3% of all cancer-related deaths) [1]. In the United States, the burden of CRC remains similarly substantial, accounting for nearly 10% of all cancer cases and approximately 8–9% of cancer-related deaths [1,2,3]. According to the Indian Council of Medical Research, the annual incidence rates (AIRs) of colon and rectal cancer are approximately 4 per 100,000 population in both men and women [4]. Given its high incidence and substantial contribution to cancer-related mortality worldwide, CRC remains a major public health concern, emphasizing the need for effective strategies for early detection that may improve patient outcomes and survival. CRC typically develops gradually through the adenoma–carcinoma sequence or the serrated pathway [5], providing an important window of opportunity for early detection and intervention through organized screening programs. In the United States, the incidence of late-stage CRC among adults aged over 50 years decreased by approximately 2% annually between 2014 and 2018, a trend likely attributable, at least in part, to screening [2]. In Romania, CRC is the second most commonly diagnosed cancer after lung cancer, with approximately 12,000–13,000 new cases annually (accounting for approximately 13–13.3% of all newly diagnosed cancer cases) and more than 6000 deaths per year [1].

Although several CRC screening modalities are currently available, screening uptake remains below the 80% target established by the American National Colorectal Cancer Roundtable. Existing screening pathways are also associated with several limitations related to accessibility, cost, cultural acceptance, risk of complications, test performance, and knowledge gaps among both patients and healthcare providers [1,6,7,8,9]. One widely adopted strategy involves a two-step approach, in which fecal hemoglobin (Hb) is initially measured, and individuals with values above a predefined threshold are subsequently referred for colonoscopy. Early CRC screening programs relied primarily on guaiac-based fecal occult blood testing (gFOBT) [10,11]. However, because the diagnostic performance of gFOBT may be influenced by dietary factors and certain medications [11,12], many screening programs have progressively adopted fecal immunochemical testing (FIT) as the preferred stool-based screening method [3]. Compared with gFOBT, FIT is easier to perform and has demonstrated superior diagnostic performance, particularly in terms of sensitivity and specificity for CRC detection [13,14,15,16]. Reported sensitivity for CRC may reach approximately 91%, whereas sensitivity for advanced adenomas is considerably lower, with reported values of up to approximately 40% [13,14,15,16]. The implementation of FIT-based screening programs has been associated with reductions in both overall and advanced-stage CRC incidence [17,18], as well as substantial reductions in CRC-related mortality, reaching up to 62% in some studies [19].

These data have prompted many countries to implement and further optimize population-based CRC screening strategies. In the European Union, current recommendations aim to achieve screening coverage of 90% of the eligible population aged 50–74 years, with the fecal immunochemical test (FIT) recommended as the initial screening method, followed by colonoscopy in individuals with a positive result. In the United States, screening recommendations differ somewhat, with average-risk adults advised to initiate CRC screening at the age of 45 years. Available screening options include high-sensitivity stool-based tests, such as FIT, guaiac-based fecal occult blood testing (gFOBT), stool DNA-based testing and recently approved serum-based tests, as well as direct visualization methods, including colonoscopy, flexible sigmoidoscopy, and computed tomography colonography. Individuals at increased risk may require earlier initiation of screening and/or more intensive surveillance strategies [1,20,21,22,23,24].

Despite higher participation rates reported with FIT compared with gFOBT [10,14], screening uptake remains suboptimal in many countries [6], often falling below the proposed 80% participation target [13]. Nevertheless, the population-level benefits of organized CRC screening are increasingly evident. A recent international population-based study reported that countries with long-established CRC screening programs, including Denmark, the Netherlands and Slovenia, experienced some of the largest declines in CRC mortality over time [22].

Among the various CRC screening tests currently used, there are non-invasive tests (fecal immunochemical test (FIT); multitarget-stool DNA (MT-sDNA) test; blood-based biomarker assays, such as plasma methylated SEPT9 test; computed tomography colonography) and invasive tests (colonoscopy, sigmoidoscopy). Colonoscopy is more sensitive for CRC and for advanced adenomas (AAs), but it is invasive, inconvenient, more expensive and associated with complications. Non-invasive tests are preferred with respect to cost, safety and convenience, their disadvantage being the low sensitivity for AAs (42% using MT-sDNA). When studying the genes using TCGA database and by experimental verification via RT-qPCR, current CRC screening modalities include both non-invasive and invasive approaches. Non-invasive methods comprise stool-based tests, such as the fecal immunochemical test (FIT) and multitarget stool DNA (mt-sDNA) testing, as well as blood-based biomarker assays and computed tomography colonography, whereas colonoscopy and flexible sigmoidoscopy represent the main invasive endoscopic approaches. Colonoscopy provides high sensitivity for both CRC and advanced adenomas (AAs), but its widespread use as a primary screening method may be limited by its invasive nature, cost, patient inconvenience and risk of procedure-related complications. By contrast, non-invasive tests are generally more acceptable in terms of safety, convenience and accessibility, although some exhibit lower sensitivity for advanced precancerous lesions; for example, a sensitivity of approximately 42% for AAs has been reported for mt-sDNA testing [25,26].

Advances in the molecular characterization of colorectal carcinogenesis have stimulated the development of novel biomarker-based screening approaches. Analyses of The Cancer Genome Atlas (TCGA) data, complemented by experimental validation using reverse transcription–quantitative polymerase chain reaction (RT-qPCR), have identified several genes, including ABCG2, SCGN, USP2, CLDN1, and EPHX4, as potential CRC-associated biomarkers [25,26]. Increasing knowledge of the molecular alterations underlying adenomatous polyps, sessile serrated lesions (SSLs) and CRC has further supported the development of non-invasive assays designed to detect biomarkers associated with dysplasia and neoplasia.

Despite the availability of multiple screening modalities, adherence to CRC screening recommendations remains suboptimal. Analyses of the Market Scan Commercial and Medicare Supplemental databases have reported screening adherence of approximately 70%, below the 80% target established by the National Colorectal Cancer Roundtable. Emerging biomarker-based tests may help address some of the barriers associated with existing screening modalities by providing approaches that are accurate, accessible, convenient, affordable and associated with minimal risk. Among these emerging approaches, blood-based assays detecting circulating cell-free nucleic acids, particularly cell-free DNA (cfDNA), have reached an advanced stage of clinical development. Improvements in molecular detection technologies and analytical methods have enhanced their potential application to CRC screening. Stool-based molecular assays continue to evolve, while approaches based on the gut microbiome also appear promising, although their clinical utility requires further validation in large prospective screening cohorts. Ultimately, an optimal CRC screening test should combine high sensitivity and specificity for CRC and clinically relevant precursor lesions with affordability, accessibility, convenience and a low risk of adverse events. The precise role of these emerging technologies within population-based CRC screening strategies remains to be established [9,27,28,29].

Several population groups are at increased risk of developing CRC, particularly individuals with a family history of CRC, hereditary cancer syndromes, certain metabolic comorbidities, including obesity, dyslipidemia, type 2 diabetes mellitus and metabolic syndrome, as well as those exposed to lifestyle-related risk factors such as alcohol consumption and smoking [30,31,32,33,34,35,36,37,38,39]. The extent to which these risk factors should influence the initiation, frequency, and choice of CRC screening strategies continues to be investigated.

An important consideration in CRC prevention is the balance between the benefits of organized screening, particularly the potential for early detection and improved outcomes, and its possible harms, including unnecessary diagnostic procedures, complications, overdiagnosis, and subsequent interventions [40]. Accordingly, the effectiveness of a screening strategy should be evaluated not only in terms of cancer detection rates but also in relation to its potential risks and overall benefit–harm balance.

In contrast to organized population-based screening, CRC may also be diagnosed through opportunistic or symptom-driven clinical evaluation. In this pathway, diagnostic investigations, including colonoscopy, are generally initiated during routine or problem-oriented medical encounters, frequently in response to symptoms or other clinical indications. Consequently, the population undergoing opportunistic diagnostic evaluation may differ systematically from an asymptomatic screening population, introducing potential selection bias. Furthermore, symptom-driven diagnosis may be associated with detection at more advanced stages of CRC compared with organized screening.

## 2. Materials and Methods

### 2.1. Study Design

This retrospective, non-randomized, observational study included 236 patients diagnosed with colorectal neoplasia.

The primary objective was to compare two diagnostic pathways applicable to the Romanian population—FIT-based screening and opportunistic colonoscopy—with respect to the detection of CRC at an early stage. Secondary objectives were to assess whether the FIT-based screening pathway was associated with the detection of tumors with less-advanced local invasion (lower T category) and lower lymph node involvement (lower N category) compared with the opportunistic diagnostic pathway.

The two source databases were harmonized prior to statistical analysis. Variable names, coding schemes and category labels were standardized for tumor location, local tumor invasion (T category), regional lymph node involvement (N category), metastasis (M category) and overall TNM stage. Regarding disease staging, 50% of patients in Group A and 88% of those in Group B underwent surgical resection. In the remaining patients, disease extent was assessed using imaging modalities, including computed tomography (CT), magnetic resonance imaging (MRI), and positron emission tomography combined with computed tomography (PET/CT).

Group A was derived from the ROCCAS colorectal cancer screening program, which recruited participants between December 2021 and January 2024. The patients included in the present study were recruited from the South Muntenia region, although the overall ROCCAS program covered five Romanian regions: Bucharest–Ilfov, Southeast, West, South Muntenia, and Southwest Oltenia. FIT kits were distributed to eligible participants through their general practitioners, with a sample return rate of 89%.

Screening was performed using high-sensitivity quantitative fecal immunochemical tests (FITs): OC-SENSOR^®^ in the South Muntenia, Bucharest–Ilfov, Southwest Oltenia, and Southeast regions, and FOB-GOLD^®^ in the West region. A positivity threshold of ≥20 μg hemoglobin (Hb)/g feces was applied. Participants with a positive FIT result were referred for colonoscopy at a specialized center (Fundeni Clinical Institute, Bucharest, Romania), where examinations were performed by trained screening colonoscopists. Among FIT-positive participants, 49% underwent the recommended colonoscopy, and the colonoscopy completion rate was approximately 98%.

Among 1068 colonoscopies performed within the screening program, CRC was diagnosed in 115 participants; one patient was subsequently lost to follow-up, leaving 114 patients in the final analysis. All CRC cases included in Group A were asymptomatic at the time of screening, with no symptoms suggestive of colorectal disease. Within the screened cohort, the adenoma detection rate was approximately 55–56%, while the detection rate for advanced adenomas, defined as adenomas ≥ 1 cm and/or exhibiting high-grade dysplasia, was 29%. Regarding the concept of negative or unnecessary colonoscopies, of the 1068 colonoscopies, 366 (34.27%) did not identify neoplastic lesions: 181 revealed no colorectal lesions, whereas 185 identified only non-neoplastic findings [41,42,43,44,45].

Group B patients were recruited between December 2021 and January 2024 in the following clinics from Romania: Fundeni Clinical Institute—Bucharest, Coltea Clinical Hospital—Bucharest, Saint Nicolae Hospital—Pitesti, and Laurus Medical Clinic—Pitesti, with approval from every center’s Ethics Committee. In this group, out of 2972 patients who underwent an opportunistic colonoscopy (for symptoms such as rectal bleeding, bloating, abdominal pain, anemia or at personal request) with a completion rate of 97%, 122 were found to have CRC. In this case, the adenoma detection rate was 55%, with an advanced adenoma detection rate of 16%.

### 2.2. Inclusion/Exclusion Criteria

#### 2.2.1. Group A—People from the National Screening Program ROCCAS

##### Inclusion Criteria

- Age between 50 and 75 years, with a valid ID;
- Residence in one of the selected regions in Romania from the Pilot Projects: regions of South Muntenia (residence was accepted from a valid ID) or people without valid ID documents, but with residence in the specified areas with a self-written declaration);
- At least 50% of the beneficiaries of the described procedures would be from vulnerable categories as follows:
  - People from rural areas;
  - Poor people;
  - Employed people with family income smaller than the minimum wage;
  - Unemployed people, registered at the employment agency;
  - Unemployed people, unregistered at the employment agency;
  - People without medical insurance;
  - People who benefit from social assistance;
  - People who work in agriculture on their own;
  - People from foster care or who were in foster care before;
  - People who left the child protection service;
  - Homeless people;
  - People from minorities—especially Roma people;
  - People with disabilities or people with complex needs;
  - People who have kids with disabilities;
  - People coming from families with only one parent;
  - People with alcohol, drug or other toxic substance addictions;
  - People subjected to domestic violence;
  - People involved in human trafficking.

##### Exclusion Criteria

- Age outside the target range;
- Residence outside the geographical areas eligible for the screening program;
- Increased risk of CRC requiring a dedicated surveillance strategy, except for individuals with one first-degree relative or two second-degree relatives with CRC who had not previously participated in another CRC screening program;
- Signs or symptoms suggestive of colorectal disease identified or suspected during the initial assessment questionnaire. These individuals were referred to the national healthcare system for appropriate diagnostic evaluation and management.

#### 2.2.2. Group B—Opportunistic Colonoscopy Group

##### Inclusion Criteria

- Age between 50 and 75 years;
- Residence in Romania or presence in Romania at the time of colonoscopy;
- Presence of signs or symptoms suggestive of colorectal disease and/or undergoing colonoscopy at their own request.

##### Exclusion Criteria

- Beyond target age;
- Comorbidities or clinical conditions representing a contraindication to colonoscopy;
- Previous inclusion in the ROCCAS national CRC screening program.

### 2.3. Analyzed Variables

Statistical analyses were performed using R software, version 4.6.0 (R Foundation for Statistical Computing, Vienna, Austria). The primary outcome was overall TNM stage, categorized into four ordered levels (Stages I–IV), without further subdivision into substages (e.g., IIA, IIB, or IIC). The individual T, N, and M categories were analyzed as secondary outcomes.

Overall TNM stage was treated as an ordinal categorical variable, with Stage I = 1, Stage II = 2, Stage III = 3, and Stage IV = 4. Although these categories have a natural ordering according to disease severity, the numerical differences between consecutive stages cannot be assumed to represent equal increments in disease severity. Accordingly, ordinal regression was used to assess the association between the diagnostic pathway and TNM stage.

The dependent variable was overall TNM stage, whereas the explanatory variables considered in the model included diagnostic group, tumor location, and tumor type. The diagnostic group represented the main predictor of interest. A logit link function was used to determine a cumulative logit model. The same modeling approach was applied, where appropriate, to the other ordinal outcomes.

Let (Y) denote the ordinal response variable, with (J = 4) ordered categories corresponding to Stage I, Stage II, Stage III, and Stage IV, and let (X) denote the vector of explanatory variables. Among the available ordinal regression approaches, cumulative and sequential models were considered. In a cumulative model, the response is modeled through cumulative probabilities across ordered category thresholds and does not require progression through each intermediate category. By contrast, a sequential model is based on conditional probabilities of reaching a given category after passing through the preceding categories.

For overall TNM stage, the cumulative model was considered more appropriate because TNM categories represent the disease stage observed at diagnosis rather than a longitudinal sequence of transitions observed within individual patients. Thus, assignment to a more advanced stage does not imply that intermediate stages were sequentially observed. For example, the presence of distant metastasis may result in classification as Stage IV irrespective of whether intermediate stages were previously documented.

The cumulative ordinal regression model was specified as follows:

Ln ((P (Y ≤ j|X)/(P > j|X)) = αj − βj × X, with the possible values of j = 1, 2 … J − 1.

Here, (Y) denotes the ordinal outcome and (X) the predictor of interest. The term (P (Y ≤ j|X)) represents the conditional probability that the outcome falls within category (j) or any lower category. Because the overall TNM stage comprised four ordered categories (J = 4), three cumulative logits were estimated, corresponding to (j = 1, 2, 3).

The parameters αj represent category-specific intercepts (threshold or cut-point parameters) separating adjacent cumulative levels of the ordinal outcome. These parameters are required for model estimation but were not of primary inferential interest. The coefficients βj represent the effects of the predictors at the corresponding cumulative thresholds. Exponentiation of the regression coefficients provides the corresponding odds ratios (ORs), which were reported together with the inferential statistics. A non-proportional odds specification was adopted, allowing the regression coefficients βj to vary across thresholds rather than assuming a common effect across all cumulative logits.

For the T category, a sequential ordinal regression approach was considered more appropriate because increasing T categories reflect progressively greater depth of local tumor invasion through the colorectal wall and, at the most advanced level, extension into adjacent structures. Accordingly, a continuation-ratio model was used to account for the ordered and sequential nature of local tumor invasion. The model was specified as: logit (P(Y ≤ j|Y ≤ j + 1, X)/P (Y = j + 1|Y ≤ j + 1, X)) = αj − βj × X, j = 1, 2 … J − 1, and logit function is logit(p) = ln (p/1 − p).

In this formulation, the model estimates the conditional odds associated with successive levels of the ordinal outcome, given that the observation belongs to the relevant subset of adjacent or preceding categories. As in the cumulative model, category-specific regression coefficients were allowed to vary across transitions.

To further characterize the study population, a demographic analysis was performed, as summarized in Table 1. The study cohort comprised 236 patients divided into two groups: Group A included 114 patients, while Group B included 122 patients. The groups showed a similar distribution in terms of age and sex, with a median age of 66 years in both groups and comparable proportions of male and female patients.

### 2.4. Tumor Classification

Tumors were staged according to the tumor–node–metastasis (TNM) classification defined in the eighth edition of the American Joint Committee on Cancer (AJCC) Cancer Staging Manual and in accordance with the National Comprehensive Cancer Network (NCCN) guidelines. The TNM system is the standard framework for staging CRC and provides a consistent basis for prognostic assessment, clinical communication, treatment planning, and comparison of outcomes across studies and populations [46,47]. Among the available clinicopathological prognostic factors, disease stage has consistently shown a strong association with survival in CRC [48]. Moreover, variability in histopathological tumor grading further supports the use of standardized TNM staging for prognostic assessment and research purposes [49]. The TNM classification evaluates three major components: the extent of the primary tumor (T), regional lymph node involvement (N), and the presence of distant metastases (M) [50]:

- T (primary tumor) describes the depth of local tumor invasion rather than tumor size. Tis denotes carcinoma in situ (intramucosal carcinoma), T1 indicates invasion into the submucosa, T2 invasion into the muscularis propria, and T3 extension through the muscularis propria into the peri-colorectal tissues. T4 denotes invasion of the visceral peritoneum (T4a) or direct invasion of/adherence to adjacent organs or structures (T4b). TX is used when the primary tumor cannot be assessed, whereas T0 indicates no evidence of a primary tumor [50].
- N (regional lymph nodes) describes the extent of regional lymph node involvement. N0 indicates the absence of regional lymph node metastasis. N1 generally denotes metastasis in one to three regional lymph nodes and includes specific subcategories according to the number and pattern of nodal or tumor-deposit involvement. N2 denotes metastasis in four or more regional lymph nodes, with N2a corresponding to four to six positive regional lymph nodes and N2b to seven or more. NX indicates that regional lymph nodes cannot be assessed [50,51].
- M (distant metastasis) describes the presence and distribution of distant metastatic disease. M0 indicates no distant metastasis, whereas M1 denotes distant metastatic disease. M1a indicates metastasis confined to one distant organ or site without peritoneal metastasis; M1b indicates metastases involving two or more distant organs or sites without peritoneal metastasis; and M1c indicates metastasis to the peritoneal surface, with or without metastases to other sites or organs [50]. Peritoneal metastasis is associated with an unfavorable prognosis in CRC [52,53].

TNM staging simplification is useful in clinical practice when informing about survival; for example, colorectal carcinoma demonstrated a 5-year survival rate of 74% for stage I, but only 5% for stage IV [54]:

- Stage 0—Indicates carcinoma in situ. Tis, N0, and M0.
- Stage I—Localized cancer. T1–T2, N0, and M0.
- Stage II—Locally advanced cancer, subdivided into IIA (T3N0M0) and IIB/IIC (T4 variantsN0M0).
- Stage III—Locally advanced cancer, late stages. T1–T4, N1–N2, and M0.
- Stage IV—Metastatic cancer. T1–T4, N0–N2, and M1.

### 2.5. Collecting and Management of Data

Data were obtained from the database of the national CRC screening program and from the clinical databases of the participating medical centers, with approval from the Ethics Committees of all participating institutions. Data collection, harmonization, and analysis were performed by the designated study investigators in accordance with the study protocol and applicable ethical requirements.

## 3. Results

### 3.1. Descriptive Characteristics of Analyzed Cohort

The final analytical cohort comprised 236 patients with CRC (Table 1), divided into two groups according to the diagnostic pathway. Group A included 114 patients diagnosed through the ROCCAS national screening program; all had a positive FIT result and subsequently underwent colonoscopy for diagnostic confirmation. Group B comprised 122 patients who underwent colonoscopy outside the organized screening program at one of the participating medical centers. The mean age of the overall cohort was 66 years (SD, 8.9 years); 55% of patients were male, and 45% were female. All patients were reported as White (Table 1).

The analysis aimed to determine whether the FIT-based screening pathway was associated with CRC detection at an earlier stage compared with the opportunistic diagnostic pathway. Accordingly, differences between the two groups were assessed with respect to local tumor invasion (T category), regional lymph node involvement (N category), and overall TNM stage (Table 2). Because of the limited number of patients with distant metastatic disease (M1), separate statistical analysis of the M category was considered insufficiently powered to provide reliable estimates and was therefore not reported. Patients with metastatic disease were nevertheless retained in the overall study cohort and included in the overall TNM-stage classification.

Patients were categorized according to tumor location into three groups: rectum, left-sided colon (descending and sigmoid colon), and right-sided colon (cecum, ascending colon and transverse colon). For the purposes of the present analysis, transverse colon tumors were included in the right-sided colon group because of the limited number of cases in this anatomical subgroup (Table 3).

### 3.2. TNM Staging

Overall TNM stage was considered the primary outcome. In the unadjusted binary analysis, the odds of being diagnosed with early-stage disease (Stages I–II) rather than advanced-stage disease (Stages III–IV) were higher in Group A than in Group B, although the difference did not reach statistical significance (OR = 1.68, 95% CI: 0.99–2.86; *p* = 0.062). When overall TNM stage was analyzed as an ordinal outcome, patients in Group A had significantly lower odds of being diagnosed at a more advanced stage compared with those in Group B (OR = 0.48, 95% CI: 0.27–0.88; *p* = 0.018).

The cumulative ordinal regression model further characterized the association between diagnostic pathway and TNM stage. With Group B as the reference group, patients in Group A had 2.76-fold higher odds of being diagnosed with Stage I rather than Stage II–IV disease (OR = 2.76, 95% CI: 1.49–5.10; *p* = 0.001). At the second cumulative threshold, the odds of being diagnosed with Stage I–II rather than Stage III–IV disease were 2.15-fold higher in Group A (OR = 2.15, 95% CI: 1.26–3.69; *p* = 0.005). At the final threshold, corresponding to Stages I–III versus Stage IV, the estimated odds were also higher in Group A (OR = 1.97, 95% CI: 0.93–4.18), although this association did not reach statistical significance (*p* = 0.076) (Table 4).

**Figure 1:**
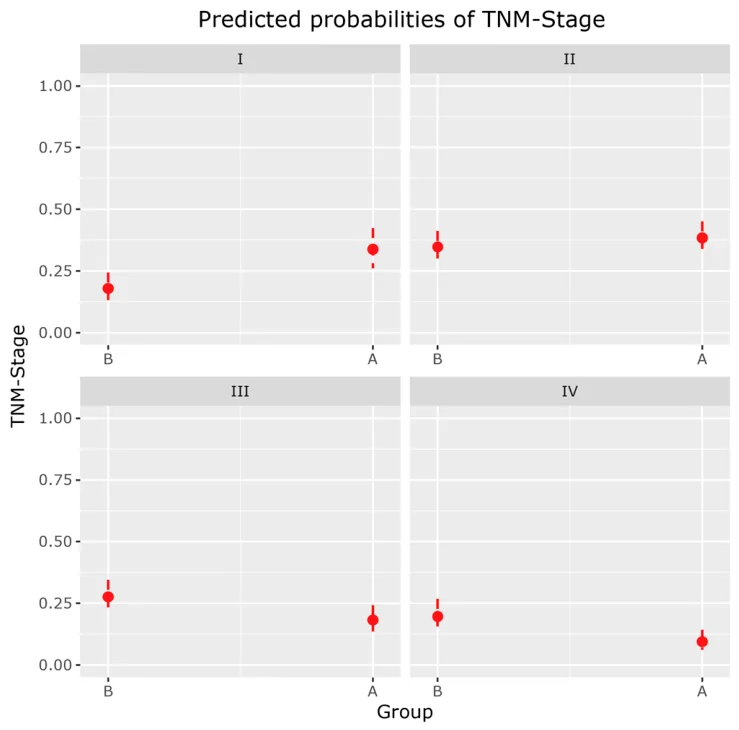
Predicted probabilities of TNM stage.

The predicted stage-specific probabilities were consistent with the cumulative-model findings (Figure 1). Compared with Group B, Group A showed a higher predicted probability of Stage I disease and a lower predicted probability of Stage III and IV disease. The predicted probability of Stage II disease was also slightly higher in Group A. Overall, the distribution of predicted probabilities indicates a shift toward earlier TNM stages among CRC cases detected through the FIT-based screening pathway compared with those diagnosed through the opportunistic diagnostic pathway.

Taken together, these analyses indicate that the FIT-based screening pathway was associated with earlier-stage CRC detection. The greatest between-group differences were observed at the lower cumulative TNM thresholds, whereas the difference for the Stage I–III versus Stage IV threshold did not reach statistical significance.

The intercepts used in Table 4 were not clinical variables (such as age or stage), but statistical model basic values, used to separate the categories of the dependent variable; they describe the reference probability—in our case, the reference Group B.

### 3.3. T Stage

**Figure 2:**
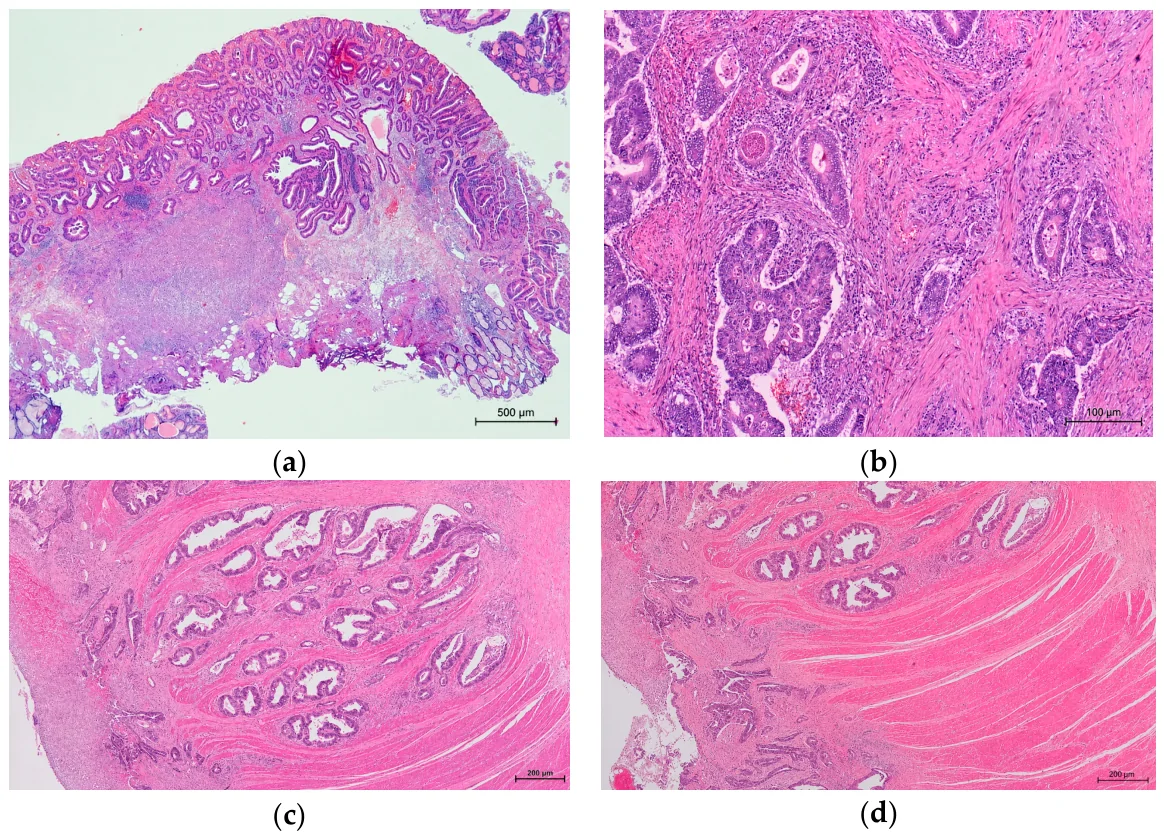
Colonic adenocarcinoma. (**a**) pT1—submucosal invasion, endoscopic submucosal dissection specimen (objective 4×, ocular lens 10×); (**b**) pT2—muscularis propria invasion (objective 20×, ocular lens 10×); (**c**) pT3—surgery (objective 10×, ocular lens 10×); (**d**) pT4—surgery (objective 10×, ocular lens 10×).

Local tumor invasion was assessed across all T categories, from T1 to T4, reflecting progressively greater depth of invasion through the colorectal wall and, in T4 disease, extension to the visceral peritoneum or adjacent organs and structures (Figure 2). The distribution of T categories according to diagnostic group is presented in Table 5.

Group A showed a substantially higher proportion of T1 tumors than Group B (25% vs. 5%) and a lower proportion of T4 tumors (11% vs. 29%), whereas T3 represented the most frequent category in both groups (49% in each group).

In the binary analysis of local tumor invasion, patients in Group A had significantly higher adjusted odds of being diagnosed with T1–T2 rather than T3–T4 disease compared with those in Group B (aOR = 2.98, 95% CI: 1.48–5.98; *p* = 0.002). Consistent with this finding, ordinal analysis showed that Group A had significantly lower odds of being diagnosed within a more advanced T category (OR = 0.27, 95% CI: 0.15–0.51; *p* < 0.001).

The cumulative ordinal regression model further characterized the association between diagnostic pathway and depth of local tumor invasion, with Group B serving as the reference group. At the first cumulative threshold, patients in Group A had 6.34-fold higher odds of being diagnosed with T1 rather than T2–T4 disease (OR = 6.34, 95% CI: 2.13–18.93; *p* = 0.001). At the second threshold, the odds of being diagnosed with T1–T2 rather than T3–T4 disease were also higher in Group A, although the association did not reach statistical significance (OR = 1.79, 95% CI: 0.98–3.25; *p* = 0.058). At the third threshold, patients in Group A had significantly higher odds of being diagnosed with T1–T3 rather than T4 disease (OR = 3.13, 95% CI: 1.55–6.28; *p* = 0.001) (Table 6).

**Figure 3:**
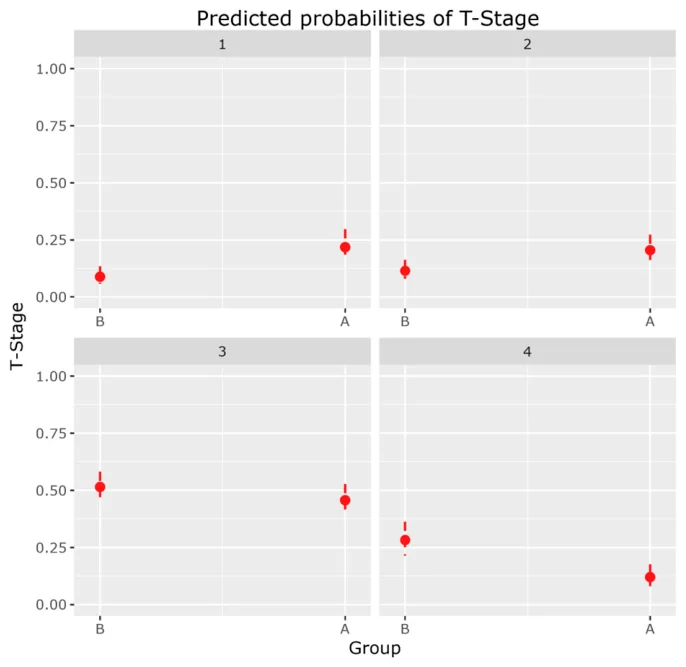
Predicted probabilities of T stage.

The predicted stage-specific probabilities were consistent with these findings (Figure 3). Compared with Group B, Group A showed a higher predicted probability of T1 disease and a markedly lower predicted probability of T4 disease. The predicted probability of T2 disease was also higher in Group A, whereas the probability of T3 disease was broadly comparable between the two groups. Overall, these findings indicate that CRC cases detected through the FIT-based screening pathway were associated with less advanced local tumor invasion compared with those diagnosed through the opportunistic diagnostic pathway.

In sensitivity analyses excluding symptom status, the association between the screening pathway and less advanced disease remained statistically significant. Group A was associated with higher adjusted odds of early overall TNM stage (aOR = 1.82, 95% CI: 1.03–3.19; *p* = 0.038), T1–T2 disease (aOR = 3.96, 95% CI: 2.15–7.29; *p* < 0.001), and M0 status (aOR = 3.47, 95% CI: 1.49–8.11; *p* = 0.004).

### 3.4. N Stage

Regional lymph node involvement was analyzed using three ordered categories (N0, N1, and N2), with their distribution according to diagnostic group presented in Table 7.

N0 disease was more frequent in Group A than in Group B (72% vs. 56%), whereas both N1 (21% vs. 28%) and N2 (7% vs. 16%) were less frequent in Group A.

The cumulative ordinal regression model further demonstrated an association between the diagnostic pathway and regional lymph node involvement. With Group B as the reference group, patients in Group A had 2.03-fold higher odds of being diagnosed with N0 rather than N1–N2 disease (OR = 2.03, 95% CI: 1.18–3.50; *p* = 0.010). At the second cumulative threshold, patients in Group A had 2.60-fold higher odds of being diagnosed with N0–N1 rather than N2 disease (OR = 2.60, 95% CI: 1.10–6.16; *p* = 0.030) (Table 8).

**Figure 4:**
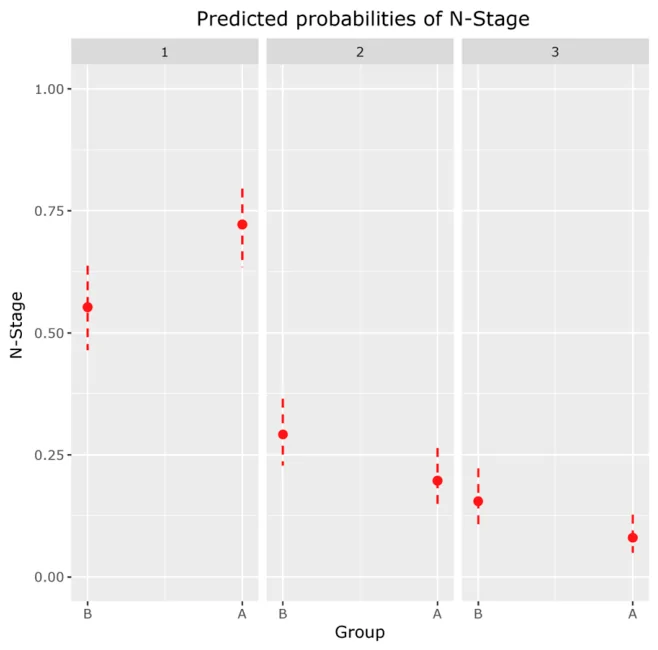
Predicted probabilities of N stage.

The predicted probabilities were consistent with these findings (Figure 4), showing a higher probability of N0 disease and lower probabilities of N1 and N2 disease in Group A compared with Group B. Overall, these results indicate that CRC cases detected through the FIT-based screening pathway were associated with less advanced regional lymph node involvement, including a greater likelihood of node-negative disease, compared with those diagnosed through the opportunistic diagnostic pathway.

## 4. Discussion

### 4.1. Study Findings

The main finding of the present study is that the FIT-based screening pathway was associated with the detection of CRC at an earlier stage compared with the opportunistic diagnostic pathway. This association was particularly evident for the extent of local tumor invasion and regional lymph node involvement, with screening-detected CRCs showing a higher likelihood of lower T categories and node-negative disease. Consistently, the overall TNM-stage distribution was shifted toward earlier disease, with a greater proportion of Stage I and II cancers detected in the screening group. When the analysis was stratified according to tumor location, statistically significant associations were maintained for left-sided colon and rectal cancers.

Importantly, these findings should not be interpreted as a direct comparison of the diagnostic accuracy of FIT and colonoscopy. Rather, the present study compares two distinct pathways leading to CRC diagnosis within a defined population: an organized FIT-based screening pathway followed by colonoscopy for individuals with a positive FIT result, and an opportunistic diagnostic pathway in which colonoscopy was performed outside the organized screening program, frequently in response to symptoms or other clinical indications. The clinically relevant question is therefore whether organized screening is associated with a more favorable stage distribution at the time of CRC diagnosis. From this perspective, our findings support the value of organized FIT-based screening programs as a strategy for increasing the detection of CRC at earlier and potentially more treatable stages.

Our findings are consistent with previous population-based studies evaluating stage distribution following the implementation of FIT-based CRC screening programs. Larsen et al. [55], in a Danish population-based study including 1,359,340 individuals, evaluated changes in CRC stage distribution following the introduction of a national FIT-based screening program. The implementation of screening was associated with a substantial increase in the detection of earlier-stage cancers, particularly Stage I disease. Similarly, the IMPATTO study reported stage-specific incidence rate ratios for screened participants compared with the unscreened population of 4.6 for Stage I, 1.4 for Stages II–III, and 0.7 for Stage IV disease. During subsequent screening rounds, the incidence rate ratio for Stage IV disease decreased further to 0.3, suggesting a substantial reduction in the occurrence of metastatic CRC among screened participants [56]. Consistent findings were reported in a Dutch study of a national FIT-based screening program published in 2023, which included 19,059 CRC cases detected during two screening rounds; 67% of these cancers were diagnosed at Stage I or II [57].

Taken together, these external data are consistent with the stage shift observed in our cohort and support the association between organized FIT-based screening and earlier CRC detection. The present study extends these observations to a Romanian population and further demonstrates that the favorable stage distribution is reflected not only in overall TNM stage, but also in lower local tumor invasion and reduced regional lymph node involvement.

### 4.2. Screening Methods

Currently, several CRC screening modalities are available, each with specific advantages and limitations related to diagnostic performance, accessibility, cost, patient acceptance and healthcare system resources [58]. These modalities can be broadly categorized into non-visual screening tests (Table 9) and direct visualization tests (Table 10).

Stool-based tests, particularly FIT, which is among the most widely used modalities for CRC screening, have demonstrated favorable cost-effectiveness compared with no screening and, in some settings, with colonoscopy-based screening strategies. FIT is generally considered an accessible and relatively low-cost screening option; however, its effectiveness at the population level depends substantially on adherence to repeated testing, and its sensitivity for detecting advanced precancerous lesions remains lower than that for CRC [71,72,73,74,75,76,77,78]. Pre-analytical factors, including sample exposure to elevated temperatures and delays in sample return or processing, may also affect FIT performance and should be considered when implementing screening programs [76]. False-positive FIT results may occur, and several factors have been associated with an increased likelihood of false positivity, including male sex, colorectal inflammatory conditions, the presence of multiple non-advanced adenomas and, in some studies, upper gastrointestinal malignancies [79].

Stool-based DNA tests have also shown favorable cost-effectiveness under specific screening scenarios, although their economic performance depends on factors such as test cost, screening interval, adherence, and comparator strategy [28,72,75,80,81]. Blood-based assays represent an emerging and potentially more acceptable approach to CRC screening and may improve screening participation because of their convenience. However, an important limitation of currently available blood-based strategies is their comparatively lower sensitivity for early-stage CRC and, particularly, for advanced precancerous lesions. Consequently, although blood-based screening may provide substantial benefits compared with no screening, its long-term effectiveness and optimal position within existing CRC screening pathways continue to be evaluated [9,64,65,75,82,83].

Whether colonoscopy should be considered the optimal primary screening modality for CRC remains a matter of debate. Although colonoscopy provides high diagnostic sensitivity and enables simultaneous detection and removal of precancerous lesions, its use as the sole population-based screening strategy is limited by invasiveness, resource requirements, costs, procedure-related risks and variable patient acceptance. Population-level reductions in CRC incidence and mortality therefore depend not only on the diagnostic performance of an individual test, but also on screening accessibility, participation, adherence and completion of the entire screening pathway [84,85,86]. Importantly, colonoscopy remains an essential component of most CRC screening strategies, particularly as the diagnostic and therapeutic follow-up procedure after a positive non-invasive screening test.

Computed tomography (CT) colonography represents an alternative structural screening modality and has demonstrated favorable cost-effectiveness in several analyses, with lower costs and fewer procedure-related complications than colonoscopy in selected settings [87,88,89,90,91]. Advances in artificial intelligence and deep learning-based image reconstruction and analysis may further improve CT colonography by facilitating image interpretation and enabling reductions in radiation exposure while maintaining adequate image quality [92].

Overall, CRC screening strategies provide substantial health benefits compared with no screening, including reductions in CRC incidence and mortality and gains in life-years. However, the magnitude of these benefits, associated harms, and cost-effectiveness varies according to the screening modality, screening interval, age at initiation and discontinuation, adherence, and healthcare setting. Modeling studies have suggested that organized programs based on biennial FIT, including strategies initiated at younger ages, as well as appropriately scheduled colonoscopy-based strategies, may be cost-effective [93,94,95]. The optimal strategy should therefore be selected by balancing expected benefits against potential harms, resource requirements, costs, and achievable population participation rather than on the basis of test performance alone.

More individualized approaches to CRC screening have also been proposed. Risk-stratified strategies incorporating polygenic risk scores, alone or in combination with established screening modalities such as FIT and colonoscopy, may help identify individuals who could benefit from earlier or more intensive screening. Such approaches offer the potential to optimize screening according to individual CRC risk, although their incremental clinical benefit, feasibility, and cost-effectiveness require further evaluation before widespread implementation [15].

An important challenge in CRC screening is ensuring timely diagnostic follow-up after an abnormal screening result, particularly when colonoscopy must be performed outside the primary care setting. This issue is especially relevant in healthcare systems in which access to specialist services may be fragmented or limited, including the Romanian healthcare setting. Gastroenterologists therefore play a central role in completing the screening pathway, not only by providing high-quality colonoscopy and maintaining adequate adenoma detection rates, but also by ensuring appropriate bowel preparation, communicating results and recommending subsequent surveillance according to colonoscopic findings and individual patient characteristics. Strengthening collaboration between gastroenterology services and primary care may improve access to CRC screening and, importantly, facilitate timely colonoscopic follow-up after a positive screening test, particularly among populations at increased risk of incomplete screening [96,97,98].

Patient-navigation programs represent another strategy for improving completion of the screening pathway. Previous studies have evaluated structured navigation interventions involving multiple patient contacts for appointment scheduling, reminders, preparation instructions and communication of test results. In some programs, participants received approximately 9–10 navigation contacts, corresponding to an average of approximately 55 min of navigator time per patient. Such interventions have been associated with improved completion of diagnostic colonoscopy and may be particularly valuable in addressing logistical, informational and healthcare-access barriers following an abnormal screening result [99,100,101].

Although TNM staging remains fundamental for prognostic assessment and therapeutic decision-making in CRC, patients within the same anatomical stage may exhibit considerable biological and clinical heterogeneity. This has stimulated interest in integrating molecular tumor characteristics with conventional anatomical staging to improve prognostic stratification and support increasingly personalized management. The eighth edition of the AJCC Cancer Staging Manual reflects the growing relevance of molecular biomarkers in CRC, although TNM classification remains primarily anatomy-based [102].

One molecular framework of particular interest is the Consensus Molecular Subtype (CMS) classification, which categorizes CRC into four major biological subtypes: CMS1 (MSI-immune), characterized by microsatellite instability, immune activation and frequent BRAF alterations; CMS2 (canonical), characterized predominantly by WNT and MYC pathway activation; CMS3 (metabolic), associated with metabolic dysregulation and frequent KRAS alterations; CMS4 (mesenchymal), characterized by prominent TGF-β activation, stromal infiltration and mesenchymal features. These molecular subtypes differ in their biological characteristics, clinical behavior and prognostic patterns and may therefore provide information complementary to conventional TNM staging [103,104].

Associations between CMS and TNM stage have been reported, although their distribution across disease stages is heterogeneous and should not be interpreted as a direct correspondence between molecular subtype and anatomical stage. Integrating molecular classification with conventional clinicopathological parameters may contribute to improved risk stratification and potentially help identify biologically distinct tumors within the same TNM stage. However, CMS classification alone is not currently sufficient to determine adjuvant treatment, and therapeutic decisions should continue to incorporate established clinicopathological and validated molecular biomarkers.

More broadly, advances in the molecular characterization of CRC may also contribute to the future development of biomarker-based screening and early-detection strategies. Molecular alterations associated with colorectal carcinogenesis, including changes involving pathways related to mismatch repair, TGF-β signaling, apoptosis and other tumor-associated molecular processes, are being investigated as potential components of non-invasive detection approaches [105]. Nevertheless, their incorporation into population-based CRC screening requires robust validation of diagnostic performance, clinical utility, feasibility and cost-effectiveness.

### 4.3. Strengths and Limitations

This study addresses a clinically relevant question in the Romanian population using real-world, patient-level data on CRC stage at diagnosis. An important strength of the analysis is the evaluation of disease severity across complementary dimensions of the TNM classification, including overall TNM stage, depth of local tumor invasion (T category) and regional lymph node involvement (N category). In addition to clinically meaningful binary comparisons between early and advanced disease, the ordered structure of the outcomes was incorporated through ordinal regression models, allowing a more detailed assessment of stage distribution across the two diagnostic pathways. Sensitivity analyses were also performed to assess the robustness of the observed associations.

Several limitations should be acknowledged. First, the retrospective, non-randomized observational design is inherently susceptible to selection bias, residual confounding and confounding by indication. Consequently, the observed associations should not be interpreted as demonstrating a causal effect of the FIT-based screening pathway on CRC stage at diagnosis.

Second, the relatively modest sample size, particularly within some disease-stage and tumor-location subgroups, limited statistical power and the precision of some estimates. This was particularly relevant for metastatic disease and prevented more detailed subgroup analyses according to tumor location.

Third, the two diagnostic pathways involved populations recruited through different mechanisms. Participants in the FIT-based screening group were selected from an organized screening program and were asymptomatic at enrollment, whereas patients undergoing colonoscopy outside the screening program were frequently investigated because of symptoms or other clinical indications. These differences may have contributed to the observed stage distributions independently of the diagnostic pathway itself. Moreover, information on all potential pre-diagnostic confounders was not uniformly available across the two data sources, precluding comprehensive adjustment for factors that may have influenced both referral for colonoscopy and disease stage at diagnosis.

Finally, differences in the basis of disease staging should also be considered. Pathological staging following surgical resection was available for a greater proportion of patients in Group B than in Group A, whereas the remaining patients were staged primarily on the basis of imaging findings. This imbalance between pathological and clinical staging may have introduced additional heterogeneity in stage classification.

## 5. Conclusions

CRC cases detected through the FIT-based screening pathway were associated with earlier TNM stage, less advanced local tumor invasion and lower regional lymph node involvement, compared with cases diagnosed through the opportunistic pathway. Despite the modest sample size and the observational study design, these findings support the value of the ROCCAS screening program and provide further evidence for expanding organized CRC screening in Romania.

Future prospective population-based studies are needed to confirm these findings and better quantify the benefits attributable to organized screening. Further research should also evaluate complementary CRC screening approaches, including emerging blood and molecular biomarker-based tests, in the Romanian population.

## Figures and Tables

**Table 1:** Demographic data.

	Overall (N = 236)	Group A (N = 114)	Group B (N = 122)
Age, Median (IQR)	66 (59–71)	66 (62–70)	65 (57–72)
Sex, n (%)			
F	107 (45)	56 (49)	51 (42)
M	129 (55)	58 (51)	71 (58)
Resected, n (%)			
Yes	164 (69)	57 (50)	107 (88)
No	72 (31)	57 (50)	15 (12)
Location, n (%)			
Right Colon	31 (13)	18 (16)	13 (11)
Left Colon	125 (52)	64 (56)	61 (50)
Rectum	80 (34)	32 (28)	48 (39)
Vegetating tumor, n (%)			
Yes	192 (81)	78 (68)	114 (93)
No	44 (19)	36 (32)	8 (7)
TNM stage, n (%)			
1	60 (25)	40 (35)	20 (16)
2	86 (36)	41 (36)	45 (37)
3	55 (23)	21 (18)	34 (28)
4	35 (15)	12 (11)	23 (19)
N stage, n (%)			
0	150 (64)	82 (72)	68 (56)
1	58 (25)	24 (21)	34 (28)
2	28 (12)	8 (7)	20 (16)
T stage, n (%)			
1	35 (15)	29 (25)	6 (5)
2	37 (16)	16 (14)	21 (17)
3	116 (49)	56 (49)	60 (49)
4	48 (20)	13 (11)	35 (29)

Observations (236).

**Table 2:** Patients’ distribution regarding the TNM staging.

	Group B (N = 122)	Group A (N = 114)
Stage, n (%)		
TNM 1	20 (16)	40 (35)
TNM 2	45 (37)	41 (36)
TNM 3	34 (28)	21 (18)
TNM 4	23 (19)	12 (11)

Observations (236).

**Table 3:** Tumor localization.

	pT1 (N = 35)	pT2 (N = 37)	pT3 (N = 116)	pT4 (N = 48)
Localization, n (%)				
Right Colon	2 (5)	4 (11)	18 (16)	7 (15)
Left Colon	22 (62)	14 (37)	61 (52)	28 (58)
Rectum	11 (31)	19 (51)	37 (32)	13 (27)

Observations (236).

**Table 4:** TNM staging cumulative model.

Predictors	Odds Ratio	CI	*p*
(Intercept) × 1	0.20	0.12–0.32	<0.001
(Intercept) × 2	1.14	0.80–1.63	0.469
(Intercept) × 3	4.30	2.73–6.78	<0.001
Group [A] × 1	2.76	1.49–5.10	0.001
Group [A] × 2	2.15	1.26–3.69	0.005
Group [A] × 3	1.97	0.93–4.18	0.076

Observations (236), CI—confidence interval, *p*—*p*-value.

**Table 5:** T stage distribution.

	Group B (N = 122)	Group A (N = 114)
pT, n (%)		
1	6 (5)	29 (25)
2	21 (17)	16 (14)
3	60 (49)	56 (49)
4	35 (29)	13 (11)

Observations (236).

**Table 6:** T staging cumulative model.

Predictors	Odds Ratios	CI	*p*
(Intercept) × 1	0.29	0.12–0.71	0.007
(Intercept) × 2	0.45	0.29–0.71	0.001
(Intercept) × 3	2.49	1.68–3.68	<0.001
Group [A] × 1	6.34	2.13–18.93	0.001
Group [A] × 2	1.79	0.98–3.25	0.058
Group [A] × 3	3.13	1.55–6.28	0.001

Observations (236), CI—confidence interval, *p*—*p*-value.

**Table 7:** N stage distribution.

	Group B (N = 122)	Group A (N = 114)
pN, n (%)		
0	68 (56)	82 (72)
1	34 (28)	24 (21)
2	20 (16)	8 (7)

Observations (236).

**Table 8:** N stage cumulative model.

Predictors	Odds Ratios	CI	*p*
(Intercept) × 1	1.26	0.88–1.80	0.206
(Intercept) × 2	5.10	3.16–8.24	<0.001
Group [A] × 1	2.03	1.18–3.50	0.010
Group [A] × 2	2.60	1.10–6.16	0.030

Observations (236), CI—confidence interval, *p*—*p*-value.

**Table 9:** Characteristics of selected non-visual colorectal cancer screening modalities.

Screening Modality	Specimen	Screening Interval	Specificity/Sensitivity	Advantages	Limitations
FIT [59,60]	Stool	1 y	CRC 74%/94% AAD 23%/96%	Non-invasive; low cost; widely available; no bowel preparation required	Requires repeated testing; positive results require diagnostic colonoscopy; lower sensitivity for advanced precancerous lesions
Multitarget sDNA (mt-sDNA/sDNA-RT)—Cologuard/ Cologuard Plus [61]	Stool	1–3 y *	CRC 94%/91% AAD 43%/93%	Non-invasive; high sensitivity for CRC; detects multiple molecular biomarkers; no bowel preparation required	Higher cost; lower specificity than FIT; positive results require diagnostic colonoscopy
Multitarget sRNA—Colosense [62]	Stool	Ney *	CRC 94%/88% AAD 46%/90%	Non-invasive; molecular biomarker-based approach	Screening interval not yet established; limited incorporation into major screening guidelines; lower specificity may increase false-positive results and subsequent colonoscopies
H-sG-bFOBT [63]	Stool	1 y	CRC 50–75%/ 96–98% AAD 6–17%/ 96–99%	Non-invasive; low cost; widely available	Requires dietary and/or medication considerations; repeated testing required; limited sensitivity for advanced precancerous and intermittently bleeding lesions
Bb-Shield, ColoHealth [64,65]	Stool	3 y	CRC 83%/90% AAD 13%/91%	Minimally invasive; simple blood sampling; potentially greater patient acceptability and adherence	Lower sensitivity for advanced precancerous lesions; higher cost; positive results require diagnostic colonoscopy; long-term population-level effectiveness remains under evaluation

FIT—Fecal Immuno-chemical Test; AAD—advanced adenoma detection; sDNA—stool-DNA; sRNA; Ney—not established yet; Sue—still under evaluation; H-sG-bFOBT—High-sensitive Guaiac-based Fecal Occult Blood Test; Bb-Shield—Blood-based–Shield. Diagnostic performance estimates were derived from the cited studies and should not be interpreted as direct head-to-head comparisons between screening modalities. * Screening intervals and recommendations may vary according to guideline, regulatory status, and patient risk profile.

**Table 10:** Characteristics of selected direct visualization colorectal cancer screening modalities.

Screening Modality	Screening Interval	Sensitivity/Specificity	Advantages	Limitations
Colonoscopy [66,67]	10 y	CRC and adenomas > 10 mm 89–95%/89%	Diagnostic and therapeutic; allows biopsy and polypectomy; examination of the entire colon; high sensitivity for CRC and advanced precancerous lesions	Invasive; requires bowel preparation and usually sedation; resource-intensive; small risk of bleeding and perforation
Capsule Endoscopy [68]	5 y *	CRC and adenomas > 10 mm 88%/96% adenomas > 6 mm 88%/94%	Minimally invasive; does not require sedation; visualization of the colon without conventional endoscopy	Requires extensive bowel preparation; incomplete examinations may occur; positive findings require conventional colonoscopy; no biopsy or polypectomy
CT Colonography [16,69,70]	5 y	CRC and adenomas > 10 mm 67–94%/86–98% adenomas > 6 mm 73–98%/80–93%	Minimally invasive; generally well tolerated; no sedation required; relatively low radiation exposure; may identify clinically relevant extracolonic findings	Requires bowel preparation; involves ionizing radiation; positive findings require colonoscopy; does not allow biopsy or polypectomy; incidental extracolonic findings may lead to additional investigations
Flexible Sigmoidoscopy [63]	5 y *	Assumed: CRC/adenoma 6–10 mm in distal colon and rectum 85%/87% *	Less invasive than colonoscopy; shorter procedure; generally lower cost and complication rates	Examines only the distal colon and rectum; lower sensitivity for proximal lesions; abnormal findings may require complete colonoscopy

CRC, colorectal cancer; CT, computed tomography. Diagnostic performance estimates were derived from the cited studies and may vary according to lesion size, study population, technology, and reference standard; therefore, they should not be interpreted as direct head-to-head comparisons between screening modalities. * Screening intervals and recommendations may vary according to guideline and patient risk profile.

## Data Availability

The datasets presented in this article are not readily available because all data are confidential and due to privacy and ethical restrictions in our country they cannot be made public.

## Abbreviations

The following abbreviations are used in this manuscript:

CRC	Colorectal Cancer
Hb	Hemoglobin
FIT	Fecal Immunochemical Test
T	Tumor
N	Lymph Node
M	Metastasis
AIRs	Annual Incidence Rates
gFOBT	Guaiac-Based Fecal Occult Blood Test
H-sG-bFOBT	High-sensitive Guaiac-based Fecal Occult Blood Test
DNA	Deoxyribonucleic Acid
MT-sDNA	Multitarget-Stool DNA
sDNA	Stool-DNA
sRNA	Stool- Ribonucleic Acid
AA	Advanced Adenomas
SSLs	Sessile Serrated Lesions
mRNA	Messenger Ribonucleic Acid
cfDNA	Circulating Free DNA or Cell Free DNA
ID	Identification
CI	Confidence Interval
P	P-value
OR	Odds Ratio
SM	Screening Methods
V/N-v	Visual/Non-visual
SA	Start Age
SI	Screening Interval
S/S	Sensitivity/Specificity
AAD	Advancer Adenoma Detection
Ney	Not Established Yet
Sue	Still Under Evaluation
Bd-Shield	Blood-based–Shield test
NF	Not First-Line
AI	Artificial Intelligence
RT-qPCR	Reverse Transcription–Quantitative Polymerase Chain Reaction
CMS	Consensus Molecular Subtyping
TGF	Transforming Growth Factor
NADH	Nicotinamide Adenine Dinucleotide

## References

[1] Bray F., Laversanne M., Sung H., Ferlay J., Siegel R.L., Soerjomataram I., Jemal A. (2024). Global cancer statistics 2022: GLOBOCAN estimates of incidence and mortality worldwide for 36 cancers in 185 countries. CA Cancer J. Clin..

[2] Issa I.A., Noureddine M. (2017). Colorectal cancer screening: An updated review of the available options. World J. Gastroenterol..

[3] Tariq H., Kamal M.U., Sapkota B., ElShikh F., Pirzada U.A., Pullela N., Azam S., Zhang A., Baiomi A., Abbas H. (2019). Evaluation of the combined effect of factors influencing bowel preparation and adenoma detection rates in patients undergoing colonoscopy. BMJ Open Gastroenterol..

[4] Vaid A.K., Mohapatra P.N., Desai C. (2021). Indian consensus statement on the management of metastatic colorectal cancer. Int. J. Adv. Med..

[5] Nguyen L.H., Goel A., Chung D.C. (2020). Pathways of Colorectal Carcinogenesis. Gastroenterology.

[6] Siegel R.L., Wagle N.S., Cercek A., Smith R.A., Jemal A. (2023). Colorectal cancer statistics, 2023. CA Cancer J. Clin..

[7] Fu J., Gao Y., Zhou P., Huang Y., Jiao J., Lin S., Wang Y., Guo Y. (2024). D2polyp-Net: A cross-modal space-guided network for real-time colorectal polyp detection and diagnosis. Biomed. Signal Process. Control.

[8] Selvaraj J., Umapathy S., Amarnath R.N. (2025). Artificial intelligence based real time colorectal cancer screening study: Polyp segmentation and classification using multi-house database. Biomed. Signal Process. Control.

[9] Hanna M., Dey N., Grady W.M. (2023). Emerging Tests for Noninvasive Colorectal Cancer Screening. Clinical Gastroenterology and Hepatology.

[10] Mandel J.S., Church T.R., Bond J.H., Ederer F., Geisser M.S., Mongin S.J., Snover D.C., Schuman L.M. (2000). The Effect of Fecal Occult-Blood Screening on the Incidence of Colorectal Cancer. N. Engl. J. Med..

[11] Wang X., Ribbing W.H., Phillips R.V., Wang Z., van der Laan M.J., Yin L., Blom J. (2026). Sequential invitations to FOBT screening and colorectal cancer incidence. Sci. Rep..

[12] Young G.P., Symonds E.L., Allison J.E., Cole S.R., Fraser C.G., Halloran S.P., Kuipers E.J., Seaman H.E. (2015). Advances in Fecal Occult Blood Tests: The FIT Revolution. Dig. Dis. Sci..

[13] Schreuders E.H., Ruco A., Rabeneck L., Schoen R.E., Sung J.J.Y., Young G.P., Kuipersc E.J. (2015). Colorectal cancer screening: A global overview of existing programmes. Gut.

[14] Grobbee E.J., Wisse P.H., Schreuders E.H., van Roon A., van Dam L., Zauber A.G., Lansdorp-Vogelaar I., Bramer W., Berhane S., Deeks J.J. (2022). Guaiac-based faecal occult blood tests versus faecal immunochemical tests for colorectal cancer screening in average-risk individuals. Cochrane Database Syst. Rev..

[15] Chen H., Si J., Luo C., Tian J., Xin J., Yu C., Sun D., Pei P., Yang L., Millwood I.Y. (2026). Optimizing colorectal cancer screening through polygenic risk score-based risk stratification: Evidence from a population-based cohort and screening trial. Genome Med..

[16] Imperiale T.F., Gruber R.N., Stump T.E., Emmett T.W., Monahan P.O. (2019). Performance Characteristics of Fecal Immunochemical Tests for Colorectal Cancer and Advanced Adenomatous Polyps. Ann. Intern. Med..

[17] Chiu H.M., Jen G.H.H., Wang Y.W., Fann J.C.Y., Hsu C.Y., Jeng Y.C., Yen A.M., Chiu S.Y., Chen S.L., Hsu W.F. (2021). Long-term effectiveness of faecal immunochemical test screening for proximal and distal colorectal cancers. Gut.

[18] Zorzi M., Urso E.D.L. (2023). Impact of colorectal cancer screening on incidence, mortality and surgery rates: Evidences from programs based on the fecal immunochemical test in Italy. Dig. Liver Dis..

[19] Chiu H., Chen S.L., Yen A.M., Chiu S.Y., Fann J.C., Lee Y., Pan S.L., Wu M.S., Liao C.S., Chen H.H. (2015). Effectiveness of fecal immunochemical testing in reducing colorectal cancer mortality from the One Million Taiwanese Screening Program. Cancer.

[20] Adsul P., Kanabar N., Kruse-Diehr A., Dignan M., Oliveri J.M., Paskett E.D., Daniel S.R., Renée M.F., Alexis A.M., Blasé P. (2026). Generating the evidence base for implementation strategies targeting colorectal cancer screening in the accelerating colorectal cancer screening through implementation science (ACCSIS) research projects. BMC Public Health.

[21] Chang C.T., Lim X.J., Chew C.C., Mustapha F.I., Rajan P. (2026). Colorectal Cancer Screening in Malaysia: Understanding the iFOBT-to-Colonoscopy Gap. J. Gastrointest. Cancer.

[22] Cardoso R., Guo F., Heisser T., Hackl M., Ihle P., De Schutter H., Van Damme N., Valerianova Z., Atanasov T., Májek O. (2021). Colorectal cancer incidence, mortality, and stage distribution in European countries in the colorectal cancer screening era: An international population-based study. Lancet Oncol..

[23] Lin J.S., Perdue L.A., Henrikson N.B., Bean S.I., Blasi P.R. (2021). Screening for Colorectal Cancer. JAMA.

[24] FDA Approves Blood Test for Colon Cancer Detection. https://www.cbsnews.com/news/fda-approves-blood-test-colon-cancer-detection/#2024.

[25] Chang Y., Bai M., Liu Y., Bai K., Hu Y. (2026). Screening and validation of potential molecular markers for colorectal cancer: Based on bioinformatics analysis and machine learning. Clin. Exp. Med..

[26] Wang R., Ye Z., Wang Q., Xu R., Feng Y., Zhao N., Yan Y., Zhao Y., Lu X., Zheng X. (2025). Dynamic screening initiation using 16 plasma protein biomarkers with polygenic risk and PLCOm2012: A precision prevention framework for lung cancer. J. Transl. Med..

[27] Behrouzian F.G., Amirfakhrian R., Hosseini B.M., Gholamin M. (2025). Non-invasive colorectal cancer screening methods: Focusing on diagnostic genetic and epigenetic markers. Cancer Cell Int..

[28] Alfaro-Núñez A., Christensen S., Ellehauge J., Eriksen J.O., Andersen G., Jensen E.A. (2025). Correction: Beyond colonoscopy, faecal DNA mutation screening provides a potential and viable path to early colorectal cancer detection. Sci. Rep..

[29] Pan W., Hansen L., Lin H., Song C., Liang Y., Kirchner J. (2026). Fecal HBA mRNA as an alternative to FIT for colorectal cancer screening: A computational and clinical validation study. Sci. Rep..

[30] Elangovan A., Skeans J., Landsman M., Ali S.M.J., Elangovan A.G., Kaelber D.C., Sandhu D.S., Cooper G.S. (2021). Colorectal Cancer, Age, and Obesity-Related Comorbidities: A Large Database Study. Dig. Dis. Sci..

[31] Breau G., Ellis U. (2020). Risk Factors Associated With Young-Onset Colorectal Adenomas and Cancer: A Systematic Review and Meta-Analysis of Observational Research. Cancer Control.

[32] Stoffel E.M., Koeppe E., Everett J., Ulintz P., Kiel M., Osborne J., Williams L., Hanson K., Gruber S.B., Rozek L.S. (2018). Germline Genetic Features of Young Individuals With Colorectal Cancer. Gastroenterology.

[33] Daca Alvarez M., Quintana I., Terradas M., Mur P., Balaguer F., Valle L. (2021). The Inherited and Familial Component of Early-Onset Colorectal Cancer. Cells.

[34] Chen F.W., Sundaram V., Chew T.A., Ladabaum U. (2017). Advanced-Stage Colorectal Cancer in Persons Younger Than 50 Years Not Associated with Longer Duration of Symptoms or Time to Diagnosis. Clin. Gastroenterol. Hepatol..

[35] Todd B. (2021). Family History a Risk for Early-Onset Colorectal Cancer. AJN Am. J. Nurs..

[36] O’Sullivan D.E., Sutherland R.L., Town S., Chow K., Fan J., Forbes N., Heitman S.J., Hilsden R.J., Brenner D.R. (2022). Risk Factors for Early-Onset Colorectal Cancer: A Systematic Review and Meta-analysis. Clin. Gastroenterol. Hepatol..

[37] Patel S.G., Karlitz J.J., Yen T., Lieu C.H., Boland C.R. (2022). The rising tide of early-onset colorectal cancer: A comprehensive review of epidemiology, clinical features, biology, risk factors, prevention, and early detection. Lancet Gastroenterol. Hepatol..

[38] Therkildsen S.B., Andersen B., Tatari C.R. (2026). Attitudes and information-needs towards a risk-based optimization of colorectal cancer screening among Danish citizens: A qualitative study. Discov. Public Health.

[39] Tabesh E., Rahimi F., Soheilipour M., Tavakoli-Moghadam N., Ravankhah Z., Hosseinian S.Z., Adibi P. (2025). Evaluating quality indicators in colorectal cancer screening via fecal immunochemical tests: A five-year study from a developing country. BMC Gastroenterol..

[40] Jatoi I., Anderson W.F., Miller A.B., Brawley O.W. (2019). The history of cancer screening. Curr. Probl. Surg..

[41] Manuc M., Diculescu M., Dumitru E., Gheonea D.I., Jinga M., Ionita-Radu F., Mergeani D., Udrescu M., Manuc T.E., Cotruta B. (2024). Introducing Colorectal Cancer Screening in Romania—Preliminary Results from the Regional Pilot Programs (ROCCAS). J. Gastrointest. Liver Dis..

[42] Gheorghe C., Bunduc S. (2023). The Colorectal Cancer Screening Program in Romania—ROCCAS—Is Ready for the Implementation at National Level. J. Gastrointest. Liver Dis..

[43] Paraschiv M., Paraschiv M.L. (2023). ROCCAS II South-Muntenia—Model of good practice in general practitioner office. Medic.ro..

[44] Metodologia de Screening Pentru Cancerul Colorectal. https://legislatie.just.ro/Public/DetaliiDocumentAfis/285165.

[45] Aprobarea Metodologiei de Screening Pentru Cancerul Colorectal. https://legislatie.just.ro/Public/DetaliiDocumentAfis/285069.

[46] Wiggers T., Arends J.W., Volovics A. (1988). Regression analysis of prognostic factors in colorectal cancer after curative resections. Dis. Colon Rectum.

[47] Ness R.M., Llor X., Abbass M.A., Bishu S., Chen C.T., Cooper G., Early D.S., Friedman M., Fudman D., Giardiello F.M. (2024). NCCN Guidelines^®^ Insights: Colorectal Cancer Screening, Version 1.2024. J. Natl. Compr. Cancer Netw..

[48] Chapuis P.H., Dent O.F., Fisher R., Newland R.C., Pheils M.T., Smyth E., Colquhoun K. (1985). A multivariate analysis of clinical and pathological variables in prognosis after resection of large bowel cancer. Br. J. Surg..

[49] Blenkinsopp W.K., Stewart-Brown S., Blesovsky L., Kearney G., Fielding L.P. (1981). Histopathology reporting in large bowel cancer. J. Clin. Pathol..

[50] Rosen R.D., Sapra A. (2023). TNM Classification. StatPearls [Internet].

[51] Sapin M.R. (2007). Lymphatic system and its significance in immune processes. Morfologiia.

[52] Menon G., Cagir B. (2025). Colon Cancer. StatPearls [Internet].

[53] Lotfollahzadeh S., Kashyap S., Tsoris A., Recio-Boiles A., Babiker H.M. (2023). Rectal Cancer. StatPearls [Internet].

[54] Menon G., Ramos Santillan V. (2025). Peritoneal Surface Malignancies. StatPearls [Internet].

[55] Larsen M.B., Njor S., Ingeholm P., Andersen B. (2018). Effectiveness of Colorectal Cancer Screening in Detecting Earlier-Stage Disease—A Nationwide Cohort Study in Denmark. Gastroenterology.

[56] Vicentini M., Zorzi M., Bovo E., Mancuso P., Zappa M., Manneschi G., Mangone L., Giorgi Rossi P., Colorectal Cancer Screening IMPATTO Study Working Group (2019). Impact of screening programme using the faecal immunochemical test on stage of colorectal cancer: Results from the IMPATTO study. Int. J. Cancer.

[57] Kooyker A., de Jonge L., Toes-Zoutendijk E., Spaander M., van Vuuren H., Kuipers E., van Kemenade F., Ramakers C., Dekker E., Nagtegaal I. (2023). Colorectal Cancer Stage Distribution at First and Repeat Fecal Immunochemical Test Screening. Clin. Gastroenterol. Hepatol..

[58] Barakat A., Cajamarca S., Chang K.J. (2026). Advancements in Screening Strategies for Early-Onset Colorectal Cancer (EOCRC). Radiol. Clin. N. Am..

[59] Grosu S., Wesp P., Graser A., Maurus S., Schulz C., Knösel T., Cyran C.C., Ricke J., Ingrisch M., Kazmierczak P.M. (2021). Machine Learning–based Differentiation of Benign and Premalignant Colorectal Polyps Detected with CT Colonography in an Asymptomatic Screening Population: A Proof-of-Concept Study. Radiology.

[60] Shaukat A., Kahi C.J., Burke C.A., Rabeneck L., Sauer B.G., Rex D.K. (2021). ACG Clinical Guidelines: Colorectal Cancer Screening 2021. Am. J. Gastroenterol..

[61] Wesp P., Grosu S., Graser A., Maurus S., Schulz C., Knösel T., Fabritius M.P., Schachtner B., Yeh B.M., Cyran C.C. (2022). Deep learning in CT colonography: Differentiating premalignant from benign colorectal polyps. Eur. Radiol..

[62] Endo S., Nagata K., Utano K., Nozu S., Yasuda T., Takabayashi K., Hirayama M., Togashi K., Ohira H. (2025). Development and validation of computer-aided detection for colorectal neoplasms using deep learning incorporated with computed tomography colonography. BMC Gastroenterol..

[63] Heisser T., Kretschmann J., Hagen B., Niedermaier T., Hoffmeister M., Brenner H. (2023). Prevalence of Colorectal Neoplasia 10 or More Years After a Negative Screening Colonoscopy in 120 000 Repeated Screening Colonoscopies. JAMA Intern. Med..

[64] Summers R.M. (2016). Progress in Fully Automated Abdominal CT Interpretation. Am. J. Roentgenol..

[65] Chen Y., Huang Z., Feng L., Zou W., Kong D., Zhu D., Dai G., Zhao W., Zhang Y., Luo M. (2024). Deep Learning-Based Reconstruction Improves the Image Quality of Low-Dose CT Colonography. Acad. Radiol..

[66] Siegel R.L., Fedewa S.A., Anderson W.F., Miller K.D., Ma J., Rosenberg P.S., Jemal A. (2017). Colorectal Cancer Incidence Patterns in the United States, 1974–2013. JNCI J. Natl. Cancer Inst..

[67] Sheridan B., Rabeneck L., Toes-Zoutendijk E. (2026). Elements of screening and early diagnosis of lower GI neoplastic lesions—An overview. Best Pract. Res. Clin. Gastroenterol..

[68] Câmara C.F., Passos P.R.C., Cezar E.C.L., Filho J.N., Oliveira R.M.A., de Paiva C.Y.M., Quintela A.O., de Andrade A.F., Veras L.B. (2026). Second-generation capsule endoscopy for the detection of colorectal polyps: An updated systematic review and comparative meta-analysis of prospective studies. Colorectal Dis..

[69] Barnell E.K., Wurtzler E.M., La Rocca J., Fitzgerald T., Petrone J., Hao Y., Kang Y., Holmes F.L., Lieberman D.A. (2023). Multitarget Stool RNA Test for Colorectal Cancer Screening. JAMA.

[70] Imperiale T.F., Porter K., Zella J., Gagrat Z.D., Olson M.C., Statz S., Garces J., Lavin P.T., Aguilar H., Brinberg D. (2024). Next-Generation Multitarget Stool DNA Test for Colorectal Cancer Screening. N. Engl. J. Med..

[71] Mannucci A., Goel A. (2024). Stool and blood biomarkers for colorectal cancer management: An update on screening and disease monitoring. Mol. Cancer.

[72] Ladabaum U., Mannalithara A. (2016). Comparative Effectiveness and Cost Effectiveness of a Multitarget Stool DNA Test to Screen for Colorectal Neoplasia. Gastroenterology.

[73] Ladabaum U., Mannalithara A., Meester R.G.S., Gupta S., Schoen R.E. (2019). Cost-Effectiveness and National Effects of Initiating Colorectal Cancer Screening for Average-Risk Persons at Age 45 Years Instead of 50 Years. Gastroenterology.

[74] van den Puttelaar R., Nascimento de Lima P., Knudsen A.B., Rutter C.M., Kuntz K.M., de Jonge L., Escudero F.A., Lieberman D., Zauber A.G., Hahn A.I. (2024). Effectiveness and Cost-Effectiveness of Colorectal Cancer Screening with a Blood Test That Meets the Centers for Medicare & Medicaid Services Coverage Decision. Gastroenterology.

[75] Char S.K., Singh H., Ng K. (2026). Biomarkers for Early Detection and Monitoring of Colorectal Cancer. Gastroenterol. Clin. N. Am..

[76] Robertson D.J., Lee J.K., Boland C.R., Dominitz J.A., Giardiello F.M., Johnson D.A., Kaltenbach T., Lieberman D., Levin T.R., Rex D.K. (2017). Recommendations on Fecal Immunochemical Testing to Screen for Colorectal Neoplasia: A Consensus Statement by the US Multi-Society Task Force on Colorectal Cancer. Gastroenterology.

[77] Velásquez M.A., Bachelet V.C. (2026). A methodological analysis of systematic reviews of cost-effectiveness in screening, diagnosis, and treatment of colorectal cancer. Health Econ. Rev..

[78] Baile-Maxía S., Pimienta M., Sánchez-Ardila C., Castells A., Jover R., Ladabaum U. (2026). Systematic Review of Participation, Positivity, and Yield Over Time in Fecal Immunochemical Test-Based Organized Colorectal Cancer Screening Programs. Clin. Gastroenterol. Hepatol..

[79] Mirza I.A., Meng F.Y., Han Z., Wang P.Z., Li Y.Y., Zhang Y., Ma M.J., Zuo X.L., Li Y.Q., Zhou R.C. (2026). Risk factors associated with false-positive fecal immunochemical test results in colorectal cancer screening. Sci. Rep..

[80] Camilleri M., Dellon E.S. (2025). Year in Review: Selection of Best Papers in Gastroenterology and Hepatology 2024. Mayo Clin. Proc..

[81] Muñoz-Montecinos C., Quirland C., Maza F., Barrientos C., Ayala C., González-Browne C. (2026). Cost-effectiveness and budget impact of a fecal immunochemical test–based colorectal cancer screening program in a cancer center. BMC Health Serv. Res..

[82] Shaukat A., Ladabaum U., Kanth P., Lieberman D. (2025). AGA Clinical Practice Update on Current Role of Blood Tests for Colorectal Cancer Screening: Commentary. Clin. Gastroenterol. Hepatol..

[83] Ladabaum U., Mannalithara A., Weng Y., Schoen R.E., Dominitz J.A., Desai M., Lieberman D. (2024). Comparative Effectiveness and Cost-Effectiveness of Colorectal Cancer Screening with Blood-Based Biomarkers (Liquid Biopsy) vs Fecal Tests or Colonoscopy. Gastroenterology.

[84] Ladabaum U., Weinberg D.S., Castells A. (2026). Can Colonoscopy Still Be Promoted as the Best Choice for Colorectal Cancer Screening?. Gastroenterology.

[85] Levin T.R., Corley D.A., Jensen C.D., Schottinger J.E., Quinn V.P., Zauber A.G., Lee J.K., Zhao W.K., Udaltsova N., Ghai N.R. (2018). Effects of Organized Colorectal Cancer Screening on Cancer Incidence and Mortality in a Large Community-Based Population. Gastroenterology.

[86] Allison J.E. (2010). The best screening test for colorectal cancer is the one that gets done well. Gastrointest. Endosc..

[87] Knudsen A.B., Rutter C.M., Peterse E.F.P., Lietz A.P., Seguin C.L., Meester R.G.S., Perdue L.A., Lin J.S., Siegel R.L., Doria-Rose V.P. (2021). Colorectal Cancer Screening. JAMA.

[88] Sawhney T.G., Pyenson B.S., Rotter D., Berrios M., Yee J. (2018). Computed Tomography Colonography Less Costly Than Colonoscopy for Colorectal Cancer Screening of Commercially Insured Patients. Am Health Drug Benefits.

[89] Kriza C., Emmert M., Wahlster P., Niederländer C., Kolominsky-Rabas P. (2013). An international review of the main cost-effectiveness drivers of virtual colonography versus conventional colonoscopy for colorectal cancer screening: Is the tide changing due to adherence?. Eur. J. Radiol..

[90] Pickhardt P.J., Hassan C., Laghi A., Kim D.H. (2009). CT Colonography to Screen for Colorectal Cancer and Aortic Aneurysm in the Medicare Population: Cost-Effectiveness Analysis. Am. J. Roentgenol..

[91] Pyenson B., Pickhardt P.J., Sawhney T.G., Berrios M. (2015). Medicare cost of colorectal cancer screening: CT colonography vs. optical colonoscopy. Abdom. Imaging.

[92] Manuc M., Dutei C.A., Manuc T.E., Chifulescu A.E., Grama F.A. (2025). Could artificial intelligence-powered colonoscopies change the future of colorectal cancer screening?. World J. Gastroenterol..

[93] Jahn B., Sroczynski G., Santamaria J., Rochau U., Siebert S., Mühlberger N., Puntscher S., Ferlitsch M., Hackl M., Tilg H. (2026). Evidence-based decision analysis guiding clinical guidelines for an organized population-based screening for colorectal cancer. Best Pract. Res. Clin. Gastroenterol..

[94] Pilar M., Hoover S., Roberson J., Jones M., Subramanian S. (2026). Characteristics and Effectiveness of Patient Navigation Programs on Colorectal Cancer Screening and Follow-Up Colonoscopy Uptake: A Systematic Review. AJPM Focus.

[95] Shi J., Li Z., Liang D., Li D., He Y. (2026). The performance of FIT-based colorectal cancer screening: Results from a population-based program. Sci. Rep..

[96] Kim S.H., Prajapati D.P., Gupta S. (2024). Extending the Reach of Colorectal Screening to all Populations in the United States. Tech Innov. Gastrointest. Endosc..

[97] Mohammadi M., Mohammadnabizadeh S. (2026). Investigating predictive factors of participation in colorectal cancer screening based on the Preventive Health Model (PHM) and health literacy. BMC Prim. Care.

[98] Lin Y., Fan S., Chai W., Zheng N., Wang X., Wang Y., Chen L. (2026). Barriers and facilitators to population participation in colorectal cancer screening: An umbrella review. BMC Health Serv. Res..

[99] Calderón-Mora J., Mitchell V.E., Salaiz R., Chacon C., Shokar N.K. (2026). Patient navigation activities in a large community-based colorectal cancer screening program. BMC Prim. Care.

[100] King A.J., Liao Y., Chen T., Kanrar R., Chunara R., Margolin D., Nettleton D., Niederdeppe J. (2026). Testing the effects of segmented crowdsource-selected messages to improve intentions to follow colorectal cancer screening recommendations: Study protocol for a randomized controlled trial. BMC Public Health.

[101] Kimura A., Peck A., Bell-Brown A., Todd K., Akinsoto N.O., Fang V., Wood J., Issaka R.B. (2026). Navigation activities in an organized colorectal cancer screening program improve follow-up colonoscopy completion. Sci. Rep..

[102] Amin M.B., Greene F.L., Edge S.B., Compton C.C., Gershenwald J.E., Brookland R.K., Meyer L., Gress D.M., Byrd D.R., Winchester D.P. (2017). The Eighth Edition AJCC Cancer Staging Manual: Continuing to build a bridge from a population-based to a more “personalized” approach to cancer staging. CA Cancer J. Clin..

[103] Mani P., Mehra L., Deepak R., Tiwari A., Kumar S., R C., Dutta R., Dash N.R., Yadav R., Deo S.V.S. (2025). Evaluation of a novel surrogate panel for classifying human colorectal carcinomas according to consensus molecular subtypes. Hum. Pathol..

[104] Kantha A., Das D., Pai E., Kumar T., Pandey M. (2025). Consensus Molecular Subtypes (CMS) Classification: A progress towards Subtype-Driven treatments in colorectal cancer. World J. Surg. Oncol..

[105] Rejali L., Seifollahi A.R., Sanjabi F., Fatemi N., Asadzadeh A.H., Saeedi N.M., Ketabi M.P., Nazemalhosseini M.E., Mini E., Nobili S. (2023). Principles of Molecular Utility for CMS Classification in Colorectal Cancer Management. Cancers.

